# Interferon regulatory factors: the next prospective targets for tissue ischemia–reperfusion injury?

**DOI:** 10.3389/fimmu.2026.1782849

**Published:** 2026-04-07

**Authors:** Jie Xie, Xiaoli An, Yuzhe Gao, Kuiying Du, Huan Chen, Jianjun Zhang, Tao Chen, Jing Hou

**Affiliations:** 1 GuiZhou University Medical College, Guiyang, Guizhou, China; 2 Department of Breast Surgery, Guizhou Provincial People’s Hospital, Guiyang, Guizhou, China; 3 Research Institute of Transplant Medicine, Nankai University, Tianjin, China

**Keywords:** cardiovascular system, central nervous system, interferon regulatory factor, ischemia-reperfusion injury, kidney, liver

## Abstract

Ischemia–reperfusion injury (IRI) represents a severe form of tissue or organ damage that occurs spontaneously and passively during the reperfusion process, following abrupt pathophysiological disturbances in the internal milieu. Alleviating and preventing IRI during organ resection or transplantation has long been a prevalent and intricate challenge in this field. Interferon regulatory factors (IRFs) constitute a superfamily of transcription factors that play pivotal roles in regulating diverse cellular biological functions, encompassing immune response modulation, inflammatory reaction control, cell proliferation and differentiation, and antiviral defense. The activation of IRFs and their downstream gene regulation primarily depend upon signal stimulation by three types of interferons (IFNs), forming an IFN-IRF-target gene cascade pathway. Consequently, the expression functions induced by IRFs under each IFN stimulus can exhibit markedly distinct characteristics. Recent studies have demonstrated that biological events involving IRFs are widespread across the cardiovascular system, central nervous system, and organs including the kidneys, liver, and intestine. Intervention targeting IRFs and their associated pathways has emerged as a significant research direction for preventing organ IRI. Nevertheless, the underlying regulatory mechanisms require further investigation and elucidation. This review aims to systematically expound the regulatory mechanisms and research advances concerning interferon regulatory factors (IRFs) and their related pathways in IRI across multiple systems and organs, from pathophysiological and molecular biological perspectives. This work not only illuminates the molecular basis of functional heterogeneity among IRFs but also proposes two innovative theoretical frameworks: the “dynamic regulatory network” and the “organ–IRF axis”. Future research ought to integrate single-cell sequencing, organoid models, and artificial intelligence prediction to elucidate the dynamic regulatory network of IRFs, thereby addressing the challenges of translational bottlenecks. Furthermore, the development of organ-specific delivery systems and heightened attention to the roles of IRFs in novel cell death mechanisms will furnish crucial support for translating research findings in this domain into clinical practice.

## Introduction

**Figure 1:**
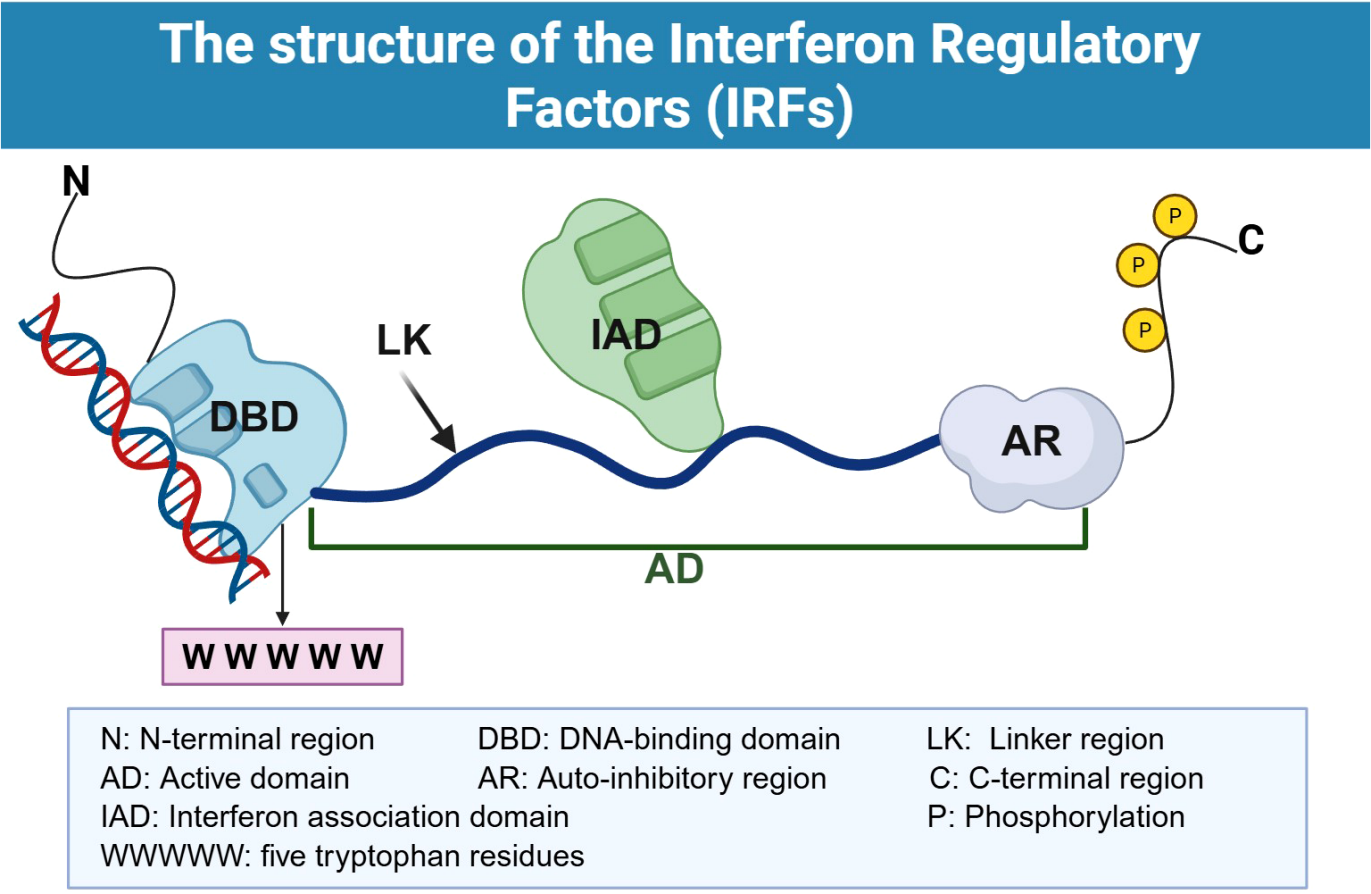
Structure of the interferon regulatory factors (IRFs). The IRF architecture comprises from the N-terminus to the C-terminus: an N-terminal DNA-binding domain (DBD) containing five conserved tryptophan residues (WWWWW) responsible for recognizing and binding to interferon-stimulated response elements (ISREs), a linker region (LK), and a C-terminal interferon association domain (IAD) together with an auto-inhibitory region (AR) constituting the activation domain (AD), wherein the IAD mediates homo- or hetero-dimerization among different IRF members and interactions with other transcription factors, while the AR participates in regulating the activation state of IRFs. The C-terminal region (C) encompasses multiple phosphorylation sites (P), with post-translational modifications exerting crucial regulatory effects upon the nuclear translocation and transcriptional activity of IRFs.

Interferon regulatory factors (IRFs) constitute a transcription factor superfamily comprising nine distinct members (IRF1 to IRF9), each encoding a unique protein (1, 2). The pivotal function of IRFs lies in transducing biological signals triggered by three classes of interferons (IFNs) produced by diverse cell types (3). Notably, the cellular sources of IFNs are remarkably heterogeneous, encompassing fibroblasts upon viral infection, dendritic cells, macrophages, and other innate immune cells, as well as activated T lymphocytes, natural killer (NK) cells and other adaptive immunity-related cells; moreover, non-immune cells such as endothelial cells and epithelial cells can also produce IFNs under stress conditions (4, 5). This signal transduction process mediated by IRFs contributes to the activation of interferon-stimulated genes (ISGs), thereby coordinating a complex regulatory network with multifaceted control capabilities (6). The IRF family exhibits a highly conserved modular architecture, which can be succinctly summarized as follows: an N-terminal DNA-binding domain (DBD), a C-terminal interferon association domain (IAD), a linker region, an auto-inhibitory region (AR), and additional post-translational modification sites (**Figure 1**) (7, 8). The DBD is consistently identified across all family members, whereas the heterogeneity within unlike DBDs is primarily responsible for the functional diversification that has been observed among the various IRFs (9). This conserved structure underscores the fundamental role of IRFs in mediating the biological responses which are initiated by IFNs and their subsequent regulation of ISGs, as indicated in a previous study (1, 2, 10). Structurally, the DBD is predominantly composed of approximately 120 amino acids that form a 5-tryptophan sequence, arranging themselves into a distinctive helix-turn-helix configuration. This structural motif is crucial for its principal function: the specific recognition and binding to the interferon-stimulated response element (ISRE) which is characterized by the consensus sequence “5′-AANNGAAA-3′” within the DNA sequence of target genes. This interaction, known as the IRFs–ISRE binding paradigm, is instrumental in modulating the expression of pertinent target genes. It is intriguing to note that, in contrast to the N-terminal DBD, there may exist certain variations within the C-terminal IRF-associated domains of the IRF family. Specifically, the domains for IRF1 and IRF2 are designated as IAD2, whereas the rest of the IRFs are characterized by the IAD1 domain (11). This divergence may arise from distinct phosphorylation patterns observed within these domains. Furthermore, additional post-translational modification sites precisely regulate biological processes of the IRF family, encompassing phosphorylation, ubiquitination, methylation, acetylation, and SUMOylation. Such domain-level variations are liable to exert influence upon the regulatory mechanisms and functional outputs of different IRFs, reflecting the intricate and nuanced roles they fulfil in cellular processes (9, 12).

**Figure 2:**
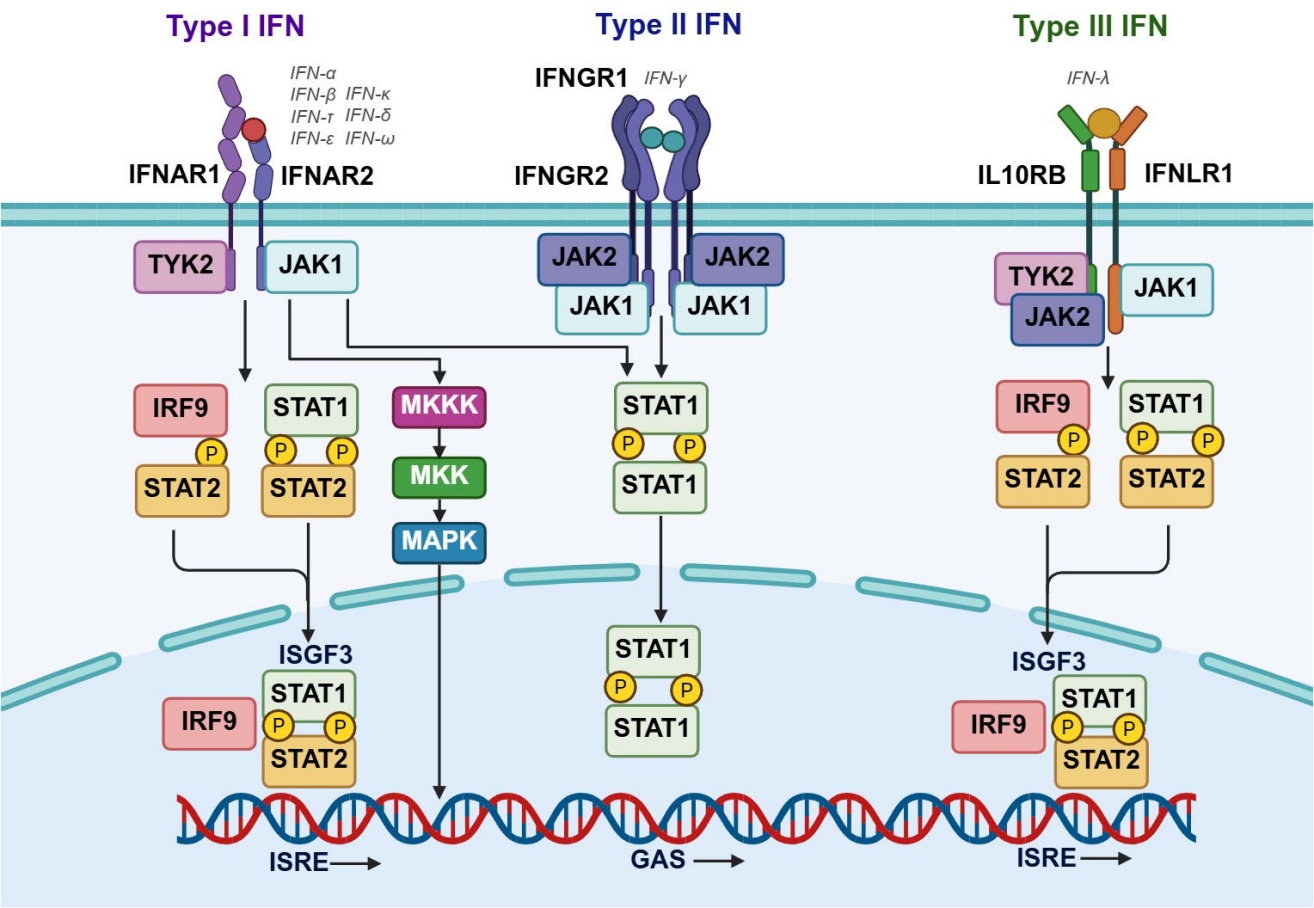
Transcriptional process and major signaling pathways of IRFs induced by interferons (IFNs). Type I IFNs (IFN-α/β and their subtypes) bind to the heterodimeric receptor IFNAR1/IFNAR2, activating the associated TYK2 and JAK1 kinases. This leads to tyrosine phosphorylation of STAT1 and STAT2 and the recruitment of IRF9 to form the heterotrimeric ISGF3 complex (STAT1-STAT2-IRF9). ISGF3 translocates to the nucleus, binds to interferon-stimulated response elements (ISREs), and induces ISG expression. Additionally, type I IFN signaling can activate the MAPK/JNK signaling pathway through the MKKK–MKK–MAPK cascade. Type II IFN pathway: IFN-γ binds as a homodimer to the IFNGR1/IFNGR2 receptor, activating JAK1 and JAK2. This triggers STAT1 phosphorylation and homodimerization, forming the gamma interferon-activated factor (GAF) complex, which binds to gamma-activated sequences (GASs) to drive target gene transcription. Type III IFNs (IFN-λ) signal through the IL10RB/IFNLR1 receptor complex, activating JAK1 and TYK2. Similar to type I IFNs, this induces STAT1/STAT2 phosphorylation and ISGF3 formation (containing IRF9). Subsequently, ISGF3 binds ISREs to initiate ISG transcription.

Overall, the transcriptional process of IRFs induced by interferons (IFNs) can be primarily categorized into three specific classifications (13): (1) JAK-STAT pathway (IFN signal amplification pathway): type I IFNs (α/β) engage with the cellular membrane IFNAR2 receptor, subsequently prompting the recruitment of IFNAR1 (14). The IFNAR receptor is widely expressed on the surface of nearly all nucleated cells, encompassing hepatocytes, cardiomyocytes, renal tubular epithelial cells, neurons, and glial cells, thereby enabling type I IFN signaling to influence the functional states of multiple tissues and organs (15, 16). This interaction stimulates the formation of a receptor complex between IFNAR-1 and IFNAR-2, thereby triggering the activation of the receptor-associated TYK2 and JAK1 kinases (17). Subsequently, the signal transduction and transcriptional activators STAT1 and STAT2 undergo tyrosine phosphorylation, and together with the recruitment of IRF9, they form a heterotrimeric transcription factor complex known as interferon-stimulated gene factor 3 (ISGF3) (18). Additionally, STAT1 homodimers, also known as IFN-γ activating factors (GAFs), are formed. These transcriptional activator complexes translocate to the cell nucleus, where they respectively activate the ISREs or γ-activated sequences (GASs) of the ISGF3 or GAF promoter elements, thereby inducing the expression of interferon-stimulated genes (ISGs) (19). Among them, IRF2 acts as a transcriptional attenuator in ISGF3-mediated transcriptional activation (20). Type I IFN signals can also induce the mitogen-activated protein kinase (MAPK)/c-Jun amino-terminal kinase (JNK) signaling pathway (2). Type II interferon (IFN-γ) binds as a homodimer, inducing the recruitment of IFN GR1 and IFN GR2 subunits, which leads to the phosphorylation of the associated JAK1 and JAK2 kinases. IFNGR is predominantly expressed on immune cells such as macrophages and T lymphocytes, yet this can also be induced on vascular endothelial cells, smooth muscle cells, and parenchymal organ cells under specific conditions, thereby mediating local inflammatory responses in tissues (21). This event subsequently triggers the phosphorylation of STAT1 and the formation of the gamma-activated factor (GAF) complex (22). (3) The signaling cascade initiated by the type III interferon receptors results in the activation of JAK1 and TYK2, subsequently inducing the recruitment of STAT1 and STAT2 to form the ISGF3 transcription factor complex (23). The type III IFN receptor (IFNLR1/IL10R2) is chiefly expressed at epithelial barrier tissues, such as the respiratory tract, intestinal tract, and hepatic biliary epithelial cells, where it fulfils distinctive roles in mucosal immunity and the maintenance of tissue homeostasis (24, 25). This complex interacts with the ISRE elements in the promoters of target genes, thereby inducing the transcription of interferon-stimulated genes (ISGs) (**Figure 2**).

As research endeavors have advanced, systematic investigations into the targeting of IRFs in animal and experimental models have elucidated the functional diversity of this family across numerous physiological and pathological processes. At the level of infectious immune regulation, IRF3 and IRF7 synergistically govern the induction of type I interferon production, with their double-knockout mice exhibiting severe innate immunodeficiency upon viral infection (4). Conversely, IRF5 primarily regulates the pro-inflammatory polarization of macrophages, and its conditional deletion markedly ameliorates pathological damage in LPS-induced sepsis (26). In the context of tumorigenesis and cellular transformation, IRF3 exerts tumor-suppressive functions in the ApcMin/+ colorectal cancer model through inhibition of the Wnt/β-catenin signaling pathway, while IRF2 blocks malignant transformation progression in the DEN-induced hepatocellular carcinoma model via the direct regulation of c-Myc transcriptional activity—both exemplifying the pivotal role of the IRF family in maintaining genomic stability (27). At the level of cell fate determination, IRF1 participates as a pro-apoptotic factor in the pathological process of myocardial ischemia–reperfusion injury, and its targeted silencing effectively reduces cell death (2). In contrast, IRF2BP2 confers protective effects on cardiomyocytes through enhancement of the autophagic flux (28). Taken together, these animal experimental evidences, derived from strategies encompassing gene knockout, conditional genetic modification, and viral vector-targeted delivery, collectively demonstrate that distinct IRF family members extensively engage in antiviral immunity, tumor suppression, and the regulation of apoptosis and autophagy through specific molecular mechanisms, thereby establishing an important preclinical theoretical foundation for comprehending the functional heterogeneity of IRFs and developing precise IRF-targeted therapeutic strategies.

**Figure 3:**
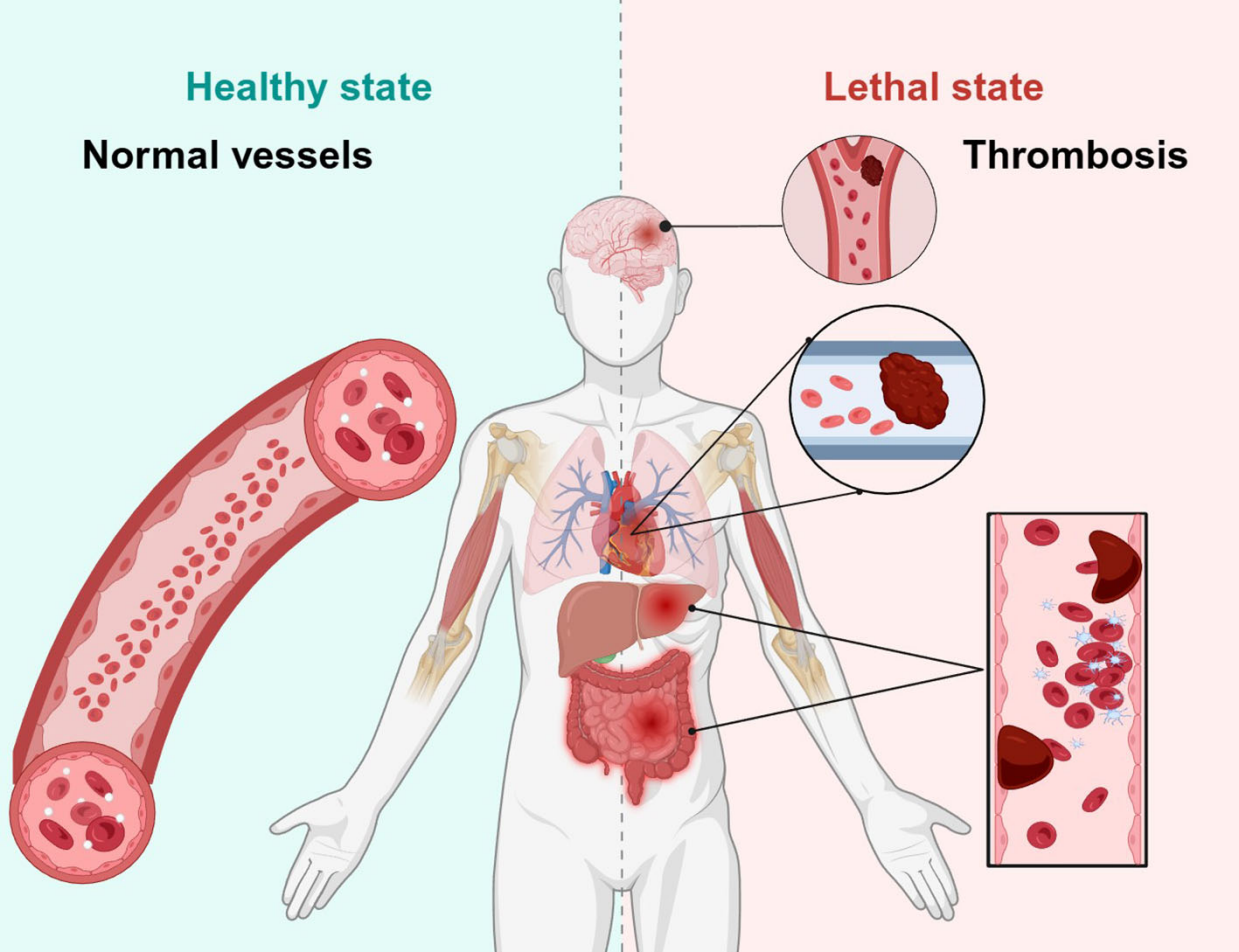
The vascular status of human organs in healthy state was compared with the thrombosis of brain, heart, liver, and intestine in the lethal state of tissue IRI.

Ischemia–reperfusion injury (IRI) represents a common form of tissue damage that typically arises during surgical procedures involving the resection of vital organs, post-transplantation operations, and the restoration of blood flow to ischemic tissues and organs caused by various diseases (29). The occurrence of IRI involves complex intercellular interactions, wherein parenchymal cells (such as hepatocytes, cardiomyocytes, renal tubular epithelial cells and neurons) constitute the primary targets of injury, while infiltrating immune cells (including neutrophils, macrophages and T lymphocytes) together with vascular endothelial cells serve as the key effector cells mediating inflammatory cascade reactions and microcirculatory disturbances (30). The pathology of IRI is predominantly driven by precipitously and typically irreversible pathophysiological alterations that are mainly launched during reperfusion within ischemic tissues (31). Common alteration includes the production of reactive oxygen species, cellular calcium overload, activation of inflammatory cells and cytokines, and especially metabolic and energetic imbalances in tissue microcirculation (32) (**Figure 3**). How to alleviate and prevent IRI during organ resection or transplantation has long been a prevalent and intricate challenge in the relevant field. Over the past few years, with the deepening of research, animal and cellular experimental models centered upon the IFNs–IRFs axis have revealed that the induction and activation of IRFs play pivotal roles in triggering IRI across diverse tissues and organs. Notably, the functions of IRFs in IRI exhibit marked cell-type specificity. In parenchymal cells, IRFs primarily regulate cell death and survival signaling (33); in immune cells, IRFs govern the production of inflammatory factors and cellular polarization (34), while in endothelial cells IRFs influence vascular permeability and the expression of adhesion molecules (35). Consequently, the targeted modulation of IRFs induced by IRI in different tissues and organs holds promise as a potential therapeutic target for eliminating IRI. In light of this, the authors and their research team shall first systematically review and summarize the distinct regulatory roles exerted by IRFs in IRI across various tissues and organs, with the aspiration of establishing a more robust theoretical foundation and research basis for future clinical investigations.

## IRF-1- mediated IRI in tissues

### IRF-1-mediated IRI in the liver

Among the IRF family, IRF-1 is currently the most well-studied transcription factor. It was discovered based on the study of the human IFN-β gene, which is expressed in most cells. Previous studies have shown that IRF-1 has important physiological functions in host cell antipathogen (36), antitumor (37), cytokine signaling (1), cell growth regulation (38), and hematopoietic cell development (39). It is noteworthy that IRF-1 knockout mice lack mature CD8+ T cells in lymphocyte and NK cell development defects, which indicates that IRF-1 plays an important role in the development and activation of immune cells (40). In addition, IRF-1 also regulates apoptosis, autophagy, and oxidative stress levels that have all been widely confirmed and emphasized in liver IRI, which makes IRF-1 serve as a potential therapeutic target for liver IRI (41). To be precise, IRF-1 has been demonstrated to be a significant mediator for liver IRI. It was irritated in the reperfusion phase with initiator participation, especially IFN-γ and other inflammation cytokines. Once the IRF-1 promoter was activated, ISGs were subsequently stimulated to interact among intra-environmental cells and amplified the injured process. On the other hand, the injured and necrotic liver cells were recognized as damage-associated molecular patterns (DAMPs), which in turn accelerated immune cell activation and IFN release, thereby broadening the rejuvenation effect of IRF-1. Interestingly, IRF-1 itself transcriptionally expressed in both hepatocyte and parenchymal cells might upregulate in abdominal aortic calcification conditions, which suggested that liver IRI was not only influenced by damaged liver cells but also merged with certain metabolic circumstances. Thus, IRF-1 possesses the ability to mediate liver IRI with interaction effect in damaged surroundings and is deemed as a promising target to lighten liver IRI (42).

During liver IRI, interferon regulatory factor-1 (IRF-1) primarily mediates its pathological effects by modulating inflammatory cascades. Experimental evidence demonstrates that IRF-1 expression undergoes significant upregulation following ischemic insult and subsequent reperfusion, thereby facilitating the transcriptional activation of multiple pro-inflammatory mediators, including interleukin-15 (IL-15) and other cytokine networks critical to the inflammatory response (43). Recent studies have demonstrated that inflammatory factors can activate hepatic innate immune cells, particularly Kupffer cells and dendritic cells, wherein activation of the High Mobility Group Box 1 (HMGB1)/Toll-like receptor 4 (TLR4) signaling pathway exacerbates liver IRI. Mechanistically, the upregulation of IRF-1 transcription facilitates its binding to the HMGB1 gene promoter, thereby inducing HMGB1 expression. The translocated HMGB1 subsequently interacts with intracellular Beclin1 to modulate the expression of autophagy-related genes such as Bcl-2, ultimately amplifying hepatocyte autophagy and aggravating liver IRI (44, 45). Notably, the nuclear translocation of HMGB1 is dependent on its high or low acetylation level, which is regulated by upstream IRF-1 binding mobilization to histone acetyltransferase p300 (HATp300) (46). Interestingly, several studies have further confirmed the positive feedback regulation mechanism between inducible nitric oxide synthase (iNOS) and IRF-1 during IRI. On the one hand, iNOS is significantly activated by various inflammatory mediators and directly interacts with the IRF-1 promoter region through the HDAC2 protein to promote its transcription; on the other hand, there is an IRF-1 binding site in the promoter region of the iNOS gene, and the activated IRF-1 gene can promote its expression by binding to the iNOS promoter (47).

**Figure 4:**
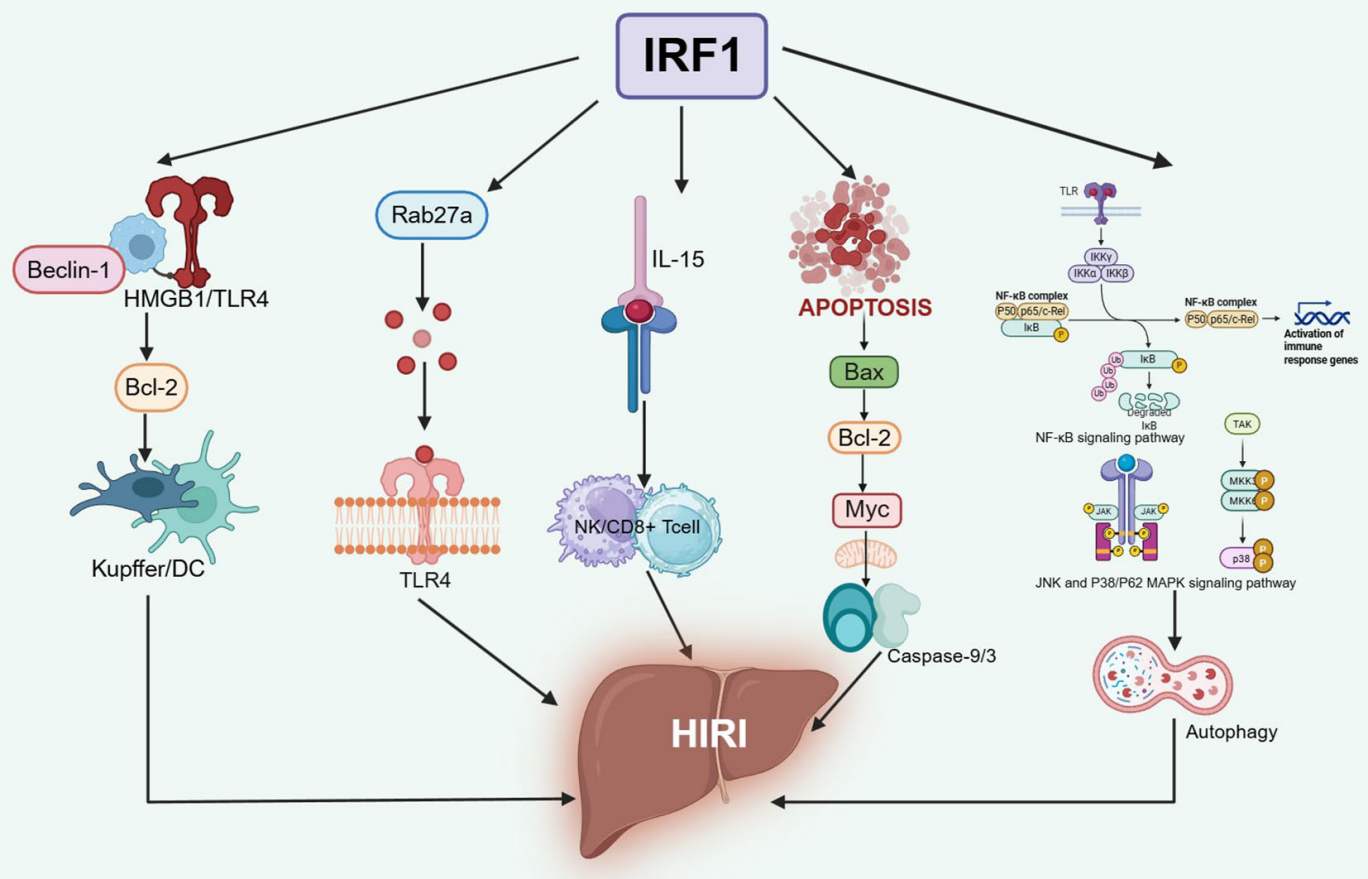
Mechanisms of IRF-1 in inducing HIRI. The increased transcription of IRF-1 regulates the transcription of Rab27a and the response to interleukin-15 (IL-15), respectively, and aggravates hepatic IRI by activating the High Mobility Group Box 1 (HMGB1)/TLR4 pathway. Meanwhile, IRF-1 overexpression significantly enhances mitochondria-dependent apoptosis by transcriptionally upregulating the pro-apoptotic protein Bax, thereby activating the cascade of Caspase-9 and Caspase-3 and exacerbating hepatic IRI. In addition, activation of the hepatocyte JNK pathway, p38/p62 MAPK, and NF-κB pathways can also trigger autophagy, which leads to more severe injury.

It is noteworthy that excessive autophagy in hepatocytes during hepatic IRI was progressively exacerbated in a time- and IRF-1-dependent manner, and activation of the JNK pathway and the P38/P62 MAPK pathway in hepatocytes could be detected by IRF-1-induced expression, triggering autophagy to a greater extent of damage (48, 49). IRF-1 overexpression significantly enhanced mitochondria-dependent apoptosis by transcriptionally regulating the expression of the pro-apoptotic protein Bax and inhibiting the function of the anti-apoptotic protein Bcl-2, leading to depolarization of the mitochondrial membrane potential and the release of cytochrome C, which then activates the cascade reaction of Caspase-9 and Caspase-3 (50). In addition, it was found that neutrophils are the main immune cell population in liver IRI, which can promote the recruitment and activation of neutrophils by secreting cytokines and chemokines during liver IRI (51). From the perspective of the potential triggering mode of neutrophil activation, IRF-1 plays a dominant role in the excretion of neutrophil extracellular vesicles. IRF-1 binds to the promoter region of Rab27a to regulate Rab27a transcription, ultimately leading to liver IRI through the neutrophil TLR-4 pathway (52). IRF-1 exacerbates liver IRI through multifaceted synergistic mechanisms, and therapeutic targeting of IRF-1 or its downstream signaling pathways, including JAK-STAT and NF-κB, may emerge as a novel strategy for liver IRI intervention (**Figure 4**).

However, critical bottlenecks persist in the current research. Existing evidence derives predominantly from murine hepatic IRI models, yet substantial species disparities exist between mouse and human livers regarding ischemic tolerance duration, immune microenvironment composition, and IRF-1 expression dynamics, thereby constraining the clinical translational value (53). A more formidable challenge lies in the fact that liver transplant recipients frequently endure combined insults of cold ischemia–reperfusion injury and warm ischemia–reperfusion injury, whereas contemporary animal models fail to recapitulate this clinical complexity (54). IRI in hepatic resection involves compensatory regeneration of the remnant liver, and the bidirectional regulatory role of IRF-1 at the transition juncture between injury and regeneration remains poorly elucidated. Consequently, future investigations must urgently transcend the limitations of conventional animal models, establishing humanized organoid-immune cell co-culture systems to reconstruct the immune-parenchymal cell interplay in hepatic IRI (55) or developing organ-on-chip technologies for real-time monitoring of the spatiotemporal activation patterns of IRF-1 (56). Simultaneously, employing single-cell sequencing to dissect the heterogeneity of IRF-1 regulatory networks across distinct hepatocyte subpopulations (such as pericentral versus periportal hepatocytes) (57) and integrating artificial intelligence to predict the interactome of IRF-1 with transcriptional cofactors and epigenetic modifying enzymes shall furnish precise targets for developing cell type-specific IRF-1-targeted strategies (58). Ultimately, through establishing prospective cohorts of liver transplant recipients, amalgamating preoperative IRF-1 expression levels, intraoperative ischemic time parameters, and postoperative hepatic functional recovery data, one may establish the clinical validity of IRF-1 as a risk-stratifying biomarker for hepatic IRI, thereby achieving a genuine transition from mechanistic investigation to precision medicine (59).

### IRF-1-mediated IRI in other tissues

In reality, IRF-1, except for being studied more extensively in liver IRI, has also been shown in current research to exhibit consistent expression changes in IRF-1 of other tissues or organs. In myocardial IRI (MIR) patients and mice model, IRF-1 was screened by using microarray experiment, and the key contributor of IRF-1 was overexpressed during MIR happening, IRF-1 knockdown did not only significantly reduced myocardial infarct size and improved left ventricular ejection fraction but mechanically inhibited cardiomyocyte apoptosis and oxidative stress level via regulating nitric oxide synthase (iNOS) promoter activity. Cardiac-specific IRF-1 knockdown significantly improved cardiac function and inhibited cardiomyocyte apoptosis after MIR injury (60). Importantly, cell ferroptosis, characterized by lipid peroxides of iron-dependent accumulation and redox disequilibrium, has been proved to mediate IRI process (61). Recent findings found that excessive expression of IRF-1 could bind to glutathione peroxidase 4 (GPX4) promoter and repressed its transcriptional activity to exacerbate ferroptosis relative myocardial dysfunction after cardiac arrest and resuscitation in a pig MIR model. Mechanistically, upstream Nrf2 could be a protector, alleviating the expression of IRF-1 (62). However, Sulforaphane (SFN), known as Nrf2 stimulator, synergistically reversed the accumulated IRF-1 expression and ultimately restored the transcription of GPX4 in cardiomyocytes after H/R. It was notable that IRF-1 seemed to be an activator for IRI via promoting severe cell ferroptosis depend on the GPX4 inhibition pattern of IRF-1-SP11 interaction or IRF-1-Zinc finger protein 350 complex (63, 64). It suggested that IRF-1 induced ferroptosis was vital for MIR injury, however, the upstream factor or complex inhibition of IRF-1 should be paid more attention for MIR prevention.

Interestingly, multi-omics analyses have revealed that IRF-1 exhibits a marked cell type-specific expression pattern in skin tissue, with particular enrichment in keratinocytes (65). This finding overturns the classical notion of IRF-1 as an “immune-exclusive transcription factor”, suggesting that skin barrier cells themselves possess the capacity to actively sense stress signals and initiate immune cascades. Studies have demonstrated that keratinocytes with high IRF-1 expression secrete pro-inflammatory factors such as IL-1α and TNF-α, forming a positive-feedback communication loop with immune cells including macrophages and T cells, thereby constituting the core driving force of the early inflammatory microenvironment following radiation injury. From the perspective of molecular triggering mechanisms of radiation stress, single high-dose irradiation (such as 20 Gy delivered in a single fraction) models the acute injury pattern seen in clinical stereotactic radiotherapy, whereas fractionated irradiation (such as 2 Gy × 10 fractions with 24-hour intervals) more closely approximates the clinical scenario of conventional fractionated radiotherapy (65). Both modalities induce uncoupling of mitochondrial oxidative phosphorylation, with mtDNA leaking from damaged mitochondria into the cytoplasm to activate the cGAS–STING innate immune recognition pathway (66). Nevertheless, critical differences exist between the two: single-dose irradiation triggers a “burst-like” release of mtDNA, resulting in rapid but transient activation of the STING-IRF-1 axis; fractionated irradiation, by contrast, generates a “low-intensity, sustained” signal through cumulative mitochondrial damage, leading to chronic activation of IRF-1 and a propensity for skin fibrosis (67, 68). This dose-pattern dependency offers novel insights for optimizing clinical radiotherapy regimens. By modulating fraction size and interfraction intervals, it may prove possible to attenuate IRF-1-mediated acute skin toxicity. Thus, IRF-1 activation is subject to multi-layered, exquisite regulation. At the post-translational modification level, the acetyltransferase p300 recruited by STING acetylates lysine residues within the nuclear localization sequence (NLS) of IRF-1, while TBK1-mediated serine phosphorylation exposes the NLS; these two processes act synergistically to promote nuclear translocation of IRF-1 (69). At the level of regulatory factors, mitochondrial single-stranded DNA binding protein 1 (SSBP1) serves as a “molecular brake”, competitively inhibiting the binding of IRF-1 to the STING/p300 complex and thereby preventing excessive inflammatory responses (70). This “activation-inhibition” dual-safeguard mechanism suggests that targeting the SSBP1-IRF-1 interaction interface may offer a more favorable safety profile than direct inhibition of IRF-1.

Of particular importance, IRF-1 governs not only conventional apoptosis but also drives pyroptosis through the Caspase-1-GSDMD axis—a form of programmed cell death endowed with dual characteristics of cellular demise and inflammatory amplification (71). IRF-1 binds directly to the Caspase-1 promoter to facilitate its transcription; activated Caspase-1 cleaves GSDMD protein, enabling its N-terminal domain to oligomerize and form pores upon the cell membrane. These pores exhibit distinctive ion selectivity: they permit passage of water molecules and IL-1β while excluding larger intracellular proteins (72). The cell swells to a critical volume through osmotic imbalance, then ruptures in an “explosive” manner, releasing DAMPs including IL-1β, IL-18 and HMGB1 (73). This “death-alarm” coupling mechanism enables the sacrifice of a single cell to recruit hundreds-fold neutrophils, generating a cascading inflammatory amplification effect. Currently, radiotherapy-associated skin injury lacks specific interventional modalities, with glucocorticoids affording merely symptomatic relief. As the central hub of “structural cell-immune dialogue”, IRF-1-targeted strategies offer dual advantages—they may block early inflammatory storms without compromising IFN signals requisite for anti-tumor immunity (74, 75). Future investigations might explore the development of IRF-1 nuclear translocation inhibitors capable of penetrating the epidermis (such as competitive peptides based upon the NLS sequence); employ nanocarriers for local delivery of SSBP1 mimetics to enhance endogenous negative regulation; and establish patient-derived radiation skin organoid models to validate individualized efficacy of IRF-1 targeting (76). This would bring tangible benefit to the millions of cancer patients receiving radiotherapy annually.

Intriguingly, recent several studies reported the unusual contribution of IRF-1 in other organs IRI. PANoptosis is a newly discovered programmed cell death pattern that involves all of apoptosis, necrosis, and pyroptosis (77). Activated in response to innate immune signaling (e.g., cGAS–STING, TLR4), IRF-1 further orchestrated PANoptosis, which not only facilitated the clearance of damaged cells but also amplified and sustained inflammatory immune response by releasing large quantities of immunogenic signals (such as DAMPs) through mechanisms including pyroptosis and necroptosis. This process drives a vicious cycle of tissue injury, thereby offering a novel therapeutic perspective for mitigating excessive inflammation following IRIs. In lung IRI model, the mRNA and protein expression level of IRF-1 and IL1A were significantly higher in IRI lung than in the control. More, in lung transplant recipients of the peripheral blood mononuclear cells, the expression of IRF-1 and IL1A were tend to be higher level and that meant those patients were predisposed candidates to suffer patients experiencing primary graft dysfunction (78). IRF-1 also played a central role as a key pro-inflammatory regulator in both renal IRI and cerebral IRI models. Studies have shown that the production of ROS induced up-regulation of IRF-1 in renal proximal tubular cells and brain tissue, which in turn activates the transcription of pro-inflammatory mediators such as CCL5, CXCL10, iNOS and TNF. Knockdown or pharmacological inhibition of IRF-1 significantly attenuated organ function damage, tissue morphology disruption and inflammatory response (79, 80). Notably, miR-130b inhibited IRF-1 expression by direct targeting in the cerebral IRI model, which not only reduced the level of downstream inflammatory factors, but also was closely related to apoptosis regulation, and ultimately ameliorated ischemia–reperfusion-induced pathological injury. This cross-organ mechanism of IRF-1 regulation provides a new perspective for understanding the common pathological process of IRI (81).

## IRF-3-mediated IRI in tissues

### IRF-3-mediated IRI in the liver

In addition to the well-established role of IRF-1 in liver IRI, recent research has brought light to the significant regulatory functions of other IRFs in liver IRI with a particular focus on the in-depth study of the regulatory mechanisms of IRF-3. IRF-3 plays an indispensable role in the modulation of type I IFN release and enabling the activation of diverse autoimmune cells to mount an early antiviral immune response. The fact that common viruses, such as HIV, SARS, and cowpox virus, have evolved to adapt and produce inhibitory proteins targeting the upstream IRF-3 gene that further underscores the significance of IRF3 in the human early antiviral response mechanisms (82). To date, upon sensing extracellular stimuli, cells utilize three intracellular initiating signal sources (RIG-I signaling, cGAS signaling, and MDA5 signaling) as sensors to perceive viruses or other stimulated signals and enable the endogenous kinases TBK1 and IKKϵ response to phosphorylate IRF-3. Ultimately, this cascade mediates genes expression associated with type I IFN (82–85).

Pioneering research on the upstream signaling pathways for IRF3-mediated liver IRI mechanism suggested that the Toll-like receptor 4 (TLR4) signaling pathway accelerated the progress of liver IRI that referred to the excessive release of inflammatory factors and accelerated liver cell damage through the downstream IRF3 pathway (86). It was mentioning that in the condition of activated TLR4–IRF3 axis, the local immune status of liver tissue was dominated by inducing CXCL10, which seemed to be a potential target for clinical liver IRI intervention (87). A latest research indicated that knockout of thioredoxin-interacting protein (TXNIP) in mouse myeloid macrophages suppressed the expression of downstream IRF3 gene; both of the liver IRI damage and hepatic inflammatory levels were significantly alleviated compared with the control (88). Several latest studies also recommended that the upstream targets’ or pathways’ inhibition of IRF3 might be a therapeutic schedule for liver IRI prevention. An experimental study identified the role of the STING signal in liver IRI, and the results displayed a strong activation of the STING-IRF3 signal, following bioinformatics prediction which confirmed that the miR-24-3p suppresses the expression of STING, thereby inhibiting the phosphorylation and activation of IRF3 and thus suppressing liver IRI injury (89). Another recent study indicated that Sirt3 mitigated liver IRI via restraining the expression and nuclear translocation of p53 and then inhibited the cGAS–STING signal and the downstream phosphorylation of IRF3 (90). Based on these findings, it is apparent that IRF3 plays a predominantly damaging role in liver IRI and stands to point that inhibiting the activation of its upstream initiating genes or key signaling pathways may also contribute to alleviate the liver IRI.

Studies have revealed that IRF3 also possesses phosphorylation- and transcription-independent activities, including IRF3-mediated apoptotic pathways. In this pathway, ubiquitinated IRF3 complexes translocate to the mitochondria together with BAX and release cytochrome c, leading to cell apoptosis (91). In a mouse model of high-fat-diet-induced hepatic injury, the non-transcription-dependent functions of IRF-3 demonstrated protective effects against liver damage (92). IRF-3 binds to IKKβ and inhibits the nuclear translocation of NF-κB, thereby reducing the expression of inflammatory factors. Such a mechanism may exert analogous effects in ischemia–reperfusion injury, alleviating hepatic damage through the suppression of inflammatory responses. Additionally, IRF3 interacts with the kinase domain of the inhibitor of NF-κB kinase β subunit (IKKβ) in the cytoplasm; this interaction prevents the phosphorylation of IKKβ, thereby limiting the release of p65 from the IKK complex and impairing the NF-κB-dependent expression of inflammatory genes (93). Thus, IRF3 itself constitutes a multifunctional signaling node and effector, whose activation can directly trigger cell death and fine-tune the intensity of inflammatory signals. This indicates that the speed and hierarchy of innate immune responses are more complex and rapid than we had imagined. Consequently, future drug development should not simply “activate” or “inhibit” IRF3 but rather require “function-specific modulation”—that is, designing molecules capable of channeling its functions toward specific pathways.

Interestingly, IRF3 deficiency may likewise exacerbate hepatic IRI, which is closely associated with excessive neutrophil activation and accelerated the release of the inflammatory mediator IL-17A. In-depth mechanistic analyses indicate that loss of IRF3 disrupts the delicate balance of the hepatic immune microenvironment. Under normal conditions, IRF3 constrains excessive neutrophil recruitment and activation through a negative feedback mechanism; when this “braking” mechanism fails, large numbers of neutrophils infiltrate the hepatic tissue and generate potent pro-inflammatory mediators such as IL-17A via degranulation and extracellular trap release. IL-17A further recruits additional inflammatory cells and amplifies oxidative stress damage, forming a vicious cycle of “inflammatory storm” (94). This finding overturns the simplified perception of IRF3 as merely a “pro-inflammatory factor”, suggesting that it may exert protective “inflammatory braking” functions in specific cell types and pathological stages, thereby providing important theoretical grounds for developing cell-type-specific IRF3 modulation strategies. Although the mechanisms by which IRF3 mediates hepatic IRI remain uncertain and controversial, future investigations must still employ advanced biotechnological approaches, including single-cell sequencing, spatial transcriptomics, and other multi-dimensional analytical methods, to conduct studies across distinct hepatocyte subpopulations—this is essential for revealing more molecular regulatory mechanisms that have yet to be elucidated.

### IRF-3-mediated IRI in other tissues

The cGAS–STING signaling activation and downstream IRF-3 phosphorylation are key, and the cGAS–STING–IRF3 axis has also made certain progress on the field of IRI in other tissues. The researchers observed a significant upregulation of IRF-3 in the cerebral IRI model, and further investigations showed that, compared to the IRF3 WT group, the cerebral infarction area and neuronal apoptosis level were significantly reduced in IRF-3 KO mice after cerebral IRI. Then, the IRF3 KO group was prone on lightening brain inflammation and oxidative stress; however, the abovementioned scenario due to IRF-3 depletion was significantly reversed once IRF3 was overexpressed (95). Additionally, the M2 polarization of the microglia instead of M1 polarization was a beneficial event for cerebral recovery after IRI. The latest research indicated that the application of a STING inhibitor could effectively alleviate brain tissue edema and neuronal damage after IRI, which also improve neurocognitive disorder in the hippocampus. Mechanistically, cerebral IRI induced the excessive release of mitochondrial DNA (mtDNA) to activate the STING signal and downstream IRF-3; NF-κB gave microglia the trend to M1 polarization (96). Analogously, MIR itself impaired protective autophagy by activating the STING-IRF3 signaling. On one side, it depended on the endoplasmic reticulum stress induction; on the other hand, the impairment of autophagy repair was disordered with the activated *Rubicon* gene, a vital autophagy inhibitor, to which IRF-3 bound its promoter region to promote its transcription (97). Another study involving a diabetic MIR model showed the cGAS–STING–IRF-3 signaling pathway that was also motivated to accelerate myocardial injury, and that was adhesively related to the excessive release of certain mtDNA in myocardial cells (98). Interestingly, a study for assessing microvascular injury after flap IRI showed that ginsenoside Rb3 was depicted to limit leukocyte–endothelial adhesion by inhibiting STING-IRF-3-mediated P-selectin activation, and ginsenoside Rb3 has been known as a cardiac IRI protector (99, 100). Based on these pioneering findings, it is self-evident that the research on tissue IRI centered on STING-IRF-3 signaling is widely unmasked and implied a promising insight for developing specific inhibitors or drugs involving the STING-IRF-3 target, which will broaden the application prospect in the field of tissue IRI protection.

**Figure 5:**
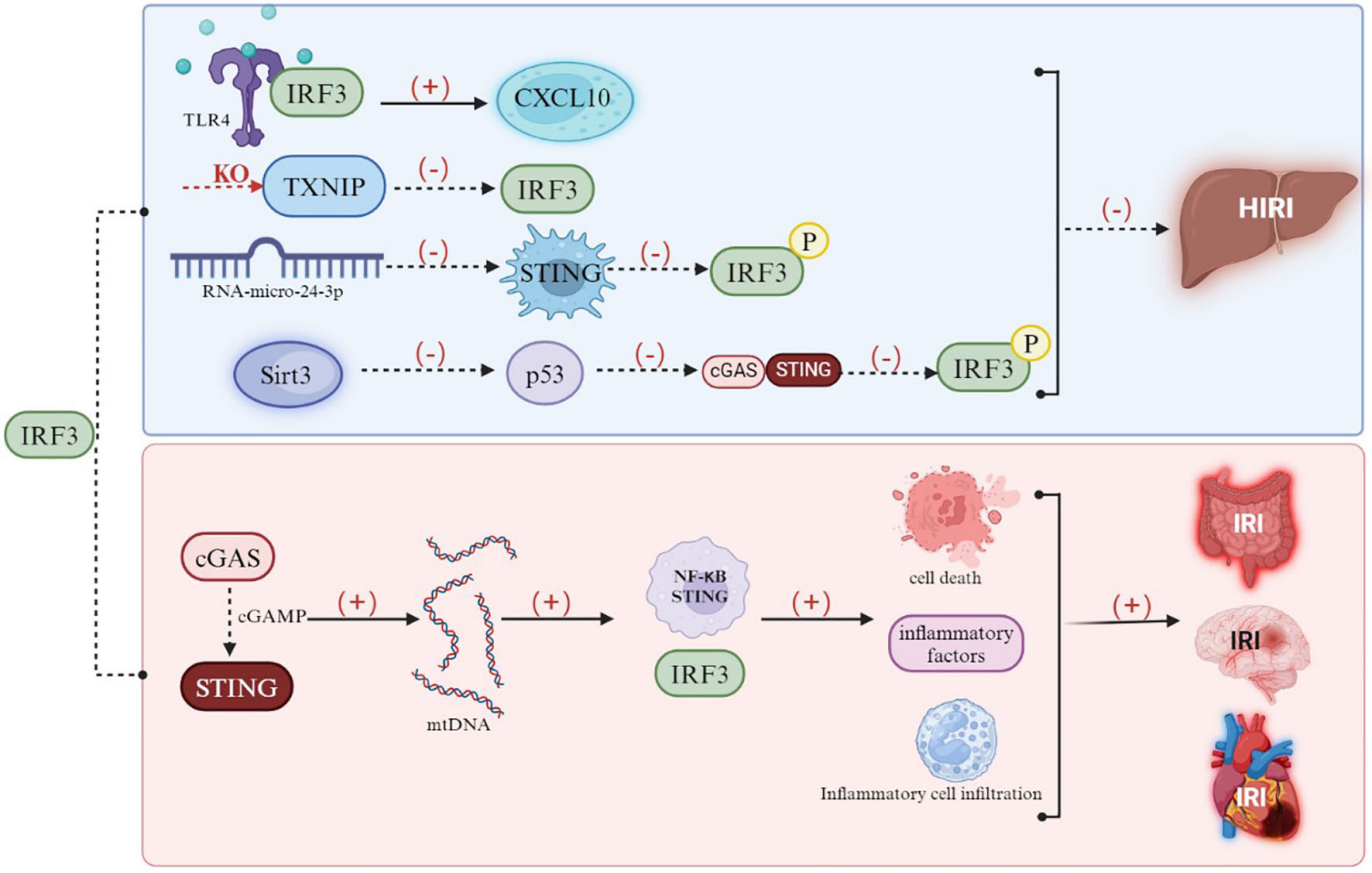
Mechanisms of IRF3 in inducing IRI. The induction of CXCL10 modulates the activated state of the TLR4–IRF3 axis, the knockout of thioredoxin-interacting protein (TXNIP) in macrophages suppresses downstream IRF3 gene expression, miR-24-3p inhibits STING expression, thereby restraining the phosphorylation and activation of IRF3, and Sirt3 attenuates hepatic IRI by suppressing p53 expression and nuclear translocation, which, in turn, inhibits the cGAS–STING signaling pathway and downstream phosphorylation of IRF3. However, in the context of IRI in other tissues, the cGAS–STING–IRF3 axis serves as a critical hub.

According to the above-mentioned past research and theories, the cGAS–STING–IRF3 signal is significantly activated to further trigger damaged patterns in the primary intestinal IRI and its secondary organ damage model (101) (**Figure 5**). Nevertheless, a recent study that referred to intestinal flora on intestinal IRI showed an unexpected observation that TLR4/TRIF/IRF-3 signaling activation led to a cascade of damaging neutrophil reactions in germ-free or antibiotic-pretreated mice, which subsequently caused the abundant formation of neutrophil extracellular traps (NETs) to exacerbate the mesenteric IRI (102). Interestingly, on the context of intestinal flora existence, the inhibition of NET formation owning to the surrounding intestinal microbiota might be conductive against mesenteric IRI (102). This result suggested that the underlying protection of either endogenous microbiota or pre-treatment with microbial agents in certain IRI might be beneficial that could be explainable by the regulation of TLR4/TRIF/IRF-3 pathway in IRI microenvironment (103). It should be pointed out that the activation of the TLR4/TRIF/IRF-3 pathway after tissue IRI was detrimental, even worsen, but advisable pre-activation of TLR4/TRIF/IRF-3 signaling before IRI seemed to be profitable in some IRI patterns (3, 104)—for example, a latest research involving LPS pre-treatment of brain IRI model showed that LPS pre-treatment surprisingly reprogrammed the TLR4/TRIF/IRF-3 pathway after brain IRI and exerted a neuroprotective effect by promoting IFNβ release (105). However, under conditions of obesity or metabolic dysregulation, sustained metabolic stress (e.g., free fatty acids and mitochondrial DNA fragments) leads to the persistent activation of IRF3, which triggers chronic inflammation and exacerbates insulin resistance—thereby establishing a pathophysiological paradigm analogous to the model of injury amplification via sustained inflammatory activation following IRI (106–108). These findings are indicative that, apart from the potential protection of microbes against intestinal IRI, reprogramming the TLR4/TRIF/IRF-3 signaling before tissue ischemia through specific stimulators could be advantageous for tissue IRI. Even if the latest findings are currently limited in specific tissues, the elaborate mechanism of IRF-3 in regulating tissue IRI still require further comprehensive exposure.

## Other tissue IRI mediated by IRFs

### Tissue IRI mediated by IRF-2

IRF-2 is a transcription factor structurally similar to IRF-1 and acts as a competitive inhibitor to compete with IRF-1 which shares the common downstream target gene DNA binding sites, thereby exerting a function opposite to that of IRF-1 (109). Studies have shown that the endogenous overexpression of IRF-2 had a potential protective effect in warm liver IRI that was achieved by limiting the release of IRF-1-dependent pro-inflammatory factors including IL-12, IFNβ, and iNOS. Similar findings were observed in heterozygous knockout IRF2 gene mice after liver transplantation (LTx) with a significant cold liver IRI (110). Notably, this mechanism is not confined to hepatic IRI but has also been observed in MIR (111), cerebral ischemic stroke reperfusion (112), and kidney IRI (113, 114), indicating a certain degree of universality. Consequently, interventional strategies targeting the IRF-1/IRF-2 homeostasis—such as developing small-molecule agents to enhance IRF-2 activity or inhibit IRF-1 function—represent a highly promising novel therapeutic direction for the treatment of IRI. Furthermore, in the research on hematopoietic stem cell (HSCT) transplantation field, it has been discovered that IRF-2 gene knockout mice often showed a loss of cell immunity function and insufficient recombination of the granulocytic series in HSCTs, indicating that IRF-2 played an essential role in maintaining the immune phenotype of HSCTs (115). In theory, due to the existing “competitive inhibitory effect” between IRF-2 and IRF-1, it is foreseeable that the regulatory effects of IRF-2 and IRF-1 on specific disease phenotypes should be “run in the opposite direction.” However, current clinical and basic research on IRF-2 in the field of organ IRI is still lacking, and the role of crucial IRF-2 upon other regulatory mechanisms in the IRI field remains to be further revealed.

## IRF-5-mediated IRI in tissues

IRF-5 is a well-studied transcription factor in the IRF family within various disease models. Similar to TLR4 signal activation and following the regulation to downstream IRF-3, IRF-5 is also induced by TLR4 signaling (116). The IRF5 mRNA transcription was launched once TLR4 was translocated and that activated IRF5-triggered hepatic IRI through the cascade of downstream pro-inflammation response. What sets IRF-5 apart is that its induction and recruitment can be activated via the TLR4/MyD88 receptor pathway compared with IRF3 (86, 117). Except for TLR4 that was deemed as IRF5 initiating signal source, a variety of kinases such as receptor-interacting protein kinase 2 (RIP2), the IKK family (TBK1, IKKα/β), and Dectin-1 are upstream initiators, which induced the phosphorylation and activation of IRF-5 (118). Targeting these mentioned upstream signaling sources for drug development or utilization may be a potential approach to suppress the activation of IRF-5 and subsequently treat liver IRI.

In addition to existing research indicating that the TLR4/MyD88/IRF-5 pathway is vital for promoting liver IRI, some studies have also suggested that IRF-5 may be a crucial regulatory key in other tissue IRI models as well. Liu and colleagues (119) studied the MIR pathogenesis based on the ventromedial hypothalamus (VMH) initiation; the results reflected the fact that TLR7/MyD88/IRF-5 signaling pathway activation led to injury and was mainly due to the stimulation of the VMH which induced sympathetic excitement. Cai and others (120), starting from the new sight of endothelial cell subpopulations, found that heart IRI activated the JAK2/STAT3 signaling pathway and induced the upregulation of IRF-5, which ultimately promoted the transcription of VCAM-1 and leukocyte adhesion inflammatory injury by IRF-5 binding to the promoter region of VCAM-1. It is worth noting that the latest research showed that by inhibiting the expression of the ligase Peli-1, E3 ubiquitin, the nuclear translocation of IRF-5, and the polarization of M1 macrophages could be effectively reduced, which significantly relieved myocardial IRI (121). In summary, modulating the expression of IRF-5 in cardiomyocytes and various cell subsets, including endothelial cell subpopulations, with a particular focus on inhibiting its nuclear translocation and the activation of associated inflammatory signaling pathways, may represent a promising therapeutic target for the treatment of cardiac IRI.

Additionally, in the context of neurological IRI, IRF-5 is also equipped with a crucial role of M1/M2 microglia/macrophages (122–124). It was found that DJ-1 impedes the interaction between p62 and TRAF6 to block the nuclear IRF-5 translocation, thereby inducing the M2 polarization of microglia/macrophages and exerting anti-inflammatory effects through microglia/macrophage subpopulations analyzed in cerebral IRI model (125). Fang et al. discovered that microRNA-22-3p could target the 5′-UTR of IRF-5 to suppress its expression and then regulated the polarization of M2 macrophages to mitigate the spinal cord IRI (126). Interestingly, unlike IRF-5 which induced M1 polarization in microglia, IRF-4 appeared to be involved in the process of M2 microglia polarization (127). This finding suggested that a combined analysis of different IRFs during tissue IRI, oriented around the IRF-4/IRF-5 balance, might be more favorable for dynamically displaying the levels of macrophage polarization in different phases (128).

Furthermore, given the diverse roles of IRF family members across different disease models, integrated monitoring and the development of multi-parameter IRF dynamic surveillance systems may prove more advantageous for precisely identifying pathogenic cell subpopulations in specific diseases and guiding individualized clinical interventions, specifically (1), utilizing single-cell RNA sequencing (scRNA-seq) combined with single-cell ATAC-seq to synchronously resolve the expression profiles and chromatin accessibility dynamics of key members including IRF1, IRF2, IRF3, IRF5, and IRF7 across distinct cell subpopulations in ischemic tissues (such as pro-inflammatory macrophages, neutrophils, and damage-associated parenchymal cells), thereby constructing an “IRF activity fingerprint” (129); (2) employing spatial barcoding technologies (such as 10× Genomics Visium) or *in situ* sequencing (ISS) to precisely localize IRF activation microdomains at the tissue section level, distinguishing IRF signaling characteristics between core injury zones and compensatory repair regions (130); (3) developing IRF1/IRF3 phosphorylation-specific antibodies or nanobodies, combined with microfluidic chip technology, to detect activated IRF fragments from peripheral blood circulating free DNA or exosomes, enabling non-invasive early warning of organ IRI (131); and (4) establishing patient-derived ischemia-sensitive organoids (such as liver buds and cardiac microtissues), integrating autologous immune cells, and real-time imaging to track the nuclear translocation kinetics of IRFs during intercellular communication, thereby providing a high-throughput platform for targeted drug screening (55). Future investigations are required to validate the clinical utility of IRF dynamic monitoring in multi-center, large-sample cohorts and to explore organ protection strategies based on IRF1/IRF3-targeted nucleic acid drugs (such as siRNA-lipid nanoparticles), thereby advancing IRF biology from fundamental research toward precision medicine.

## IRF-8-mediated IRI in tissues

The human IRF-8 gene sequence is highly conserved and located on chromosome 16 (132). Due to the weak DNA-binding affinity of IRF-8 itself, its transcriptional activity largely depends on the IRF-binding domain within its gene sequence combining with specific gene promoter DNA sequences of other transcription factors (133). Research has demonstrated that IRF8 could form heterodimers with IRF1 or IRF2, binding to the ISRE element to either inhibit the transcription of IFNs/retinoic acid-inducible genes or activate the expression of target genes (134, 135). Additionally, IRF8 heterodimers with the AP-1 factor BATF bind to the AICE site to promote gene activation in Th17 cells, B cells, and dendritic cells (136, 137). Initial consideration of IRF-8 as an important mediator was involved in the maintenance and differentiation of immune cells as well as the renewal of non-hematopoietic cells; IRF-8 also played other effects by binding to specific IFN response DNA motifs in the MHC class I antigen genes (138–141). However, more research have found that IRF-8 participated in the inhibition of the progression and clonal formation in malignant tumors, such as lung cancer, renal cell carcinoma, and soft tissue sarcomas (142).

Current research on IRF-8-mediated tissue IRI is relatively limited; its function for damage or protection is still uncertain. The latest study indicated that IRF-8 is equipped with an unexpected ability to regulate the immune-inflammatory microenvironment in the liver. Compared with wild-type mice, the IRF-8 KO mice showed significantly reduced levels of ALT/AST and markedly decreased liver necrosis after the liver IRI treatment. The underlying mechanism revealed that IRF-8 signaling activation touched off early autophagy development and the NF-kB pathway inducement, which exacerbated neutrophil infiltration and the excessive release of inflammatory factors, including CXCL1, during the liver IRI process (143). Similarly, the renal IRI model found that mice with specific IRF-8 KO exhibited significantly improved renal tubular loss and reduced renal function decline caused by renal IRI. This functional improvement was significantly interwoven with amplification of a specific subset of conventional type I dendritic cells (cDC1), which indicated that IRF-8 was an indispensable player for the differentiation of renal cDC1 subset even if the concrete mechanism was still unmasked (144). The aforementioned results indicated that in both the liver and the kidney IRI, IRF-8 was deemed as a “damager.” Nevertheless, the targeted IRF-8 KO significantly increased cell apoptosis, inflammatory levels, and the extent of oxidative stress injury in transient cerebral IRI with which specific IRF-8 transgene mice showed obvious tissue damage, oxidative stress, and inflammatory level compared to wild-type mice (145). It is apparent that the regulatory role of IRF-8 is not consistent, which may be explicable by the heterogeneity of target genes and types of microenvironment immune cells that are attributed to IRF-8 mediation in different tissue IRI models. On account of the scarcity of current basic and clinical research on IRF-8-mediated tissue IRI and translational medicine, further in-depth research on IRF-8 in this field will be more eye-catching.

## IRF-9-mediated IRI in tissues

IRF-9 usually does not exert biological functions on its own but participates in the signal transduction pathway mediated by type I IFN. IRF-9 often combines with the STAT1/STAT2 heterodimer to form the interferon-stimulated gene factor 3 (ISGF3) complex or binds directly with the STAT2 homodimer to form a “coupler”. Both complexes respond to type I IFN signaling by binding to different downstream ISGs (containing a uniform ISRE sequence) (146). IRF-9 primarily participates in immune regulation and inflammatory responses against viruses and bacteria, including the activation of NK cells, T cells, monocytes, and macrophages. Recent studies suggested that IRF-9 might refer to the biological progression of various malignancies, including neuroblastoma (147), colon carcinoma (148), and melanoma (149). However, the potential biological effect of IRF-9 and the following regulatory mechanism in tissue IRI were rarely mentioned.

**Figure 6:**
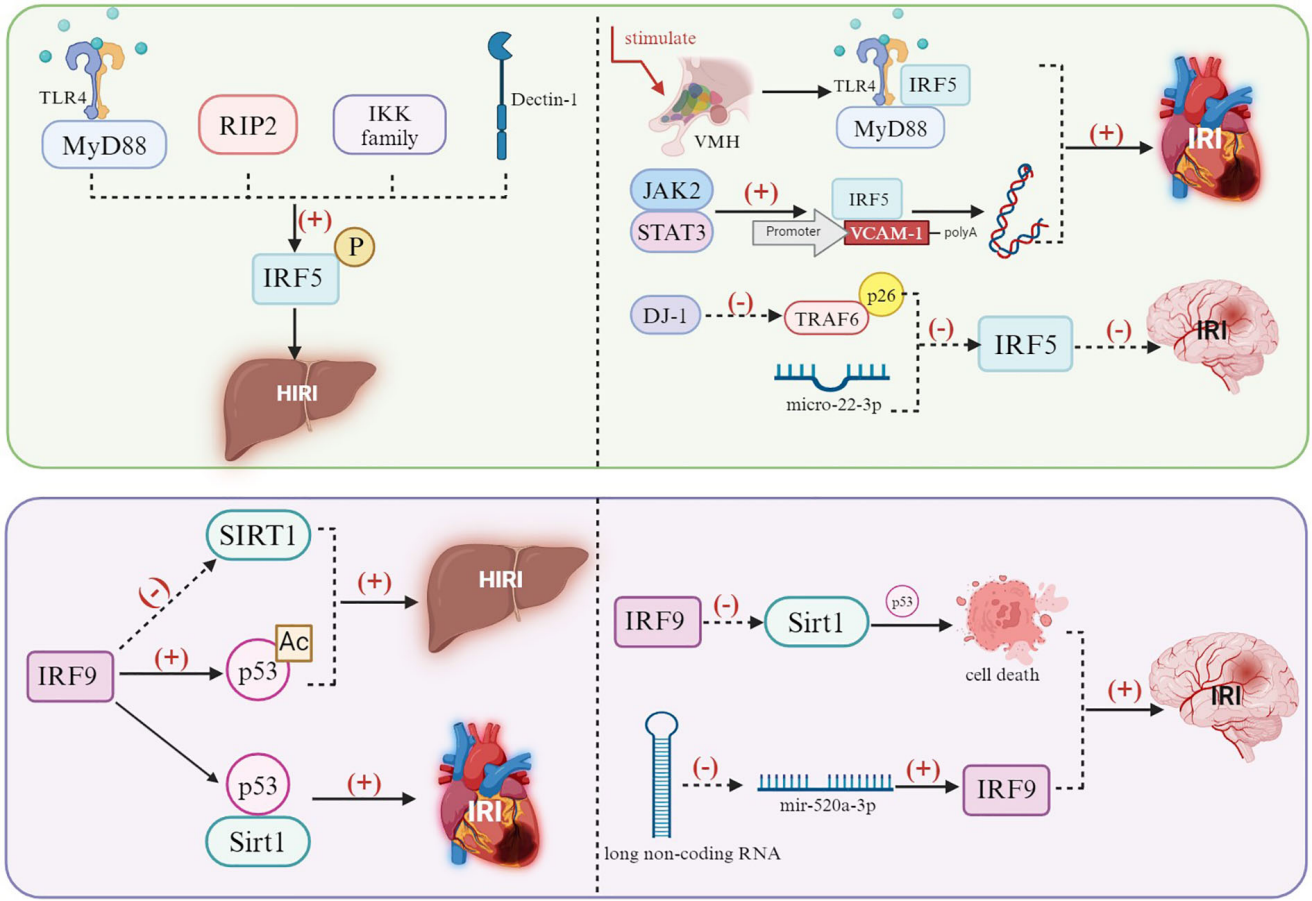
Mechanisms of IRF 5 and IRF9 in inducing IRI. Through the TLR4-mediated MyD88-dependent pathway, various kinases including receptor-interacting protein kinase 2 (RIP2), IKK family members (TBK1, IKKα/β), and Dectin-1 act as upstream initiators that induce the phosphorylation and activation of IRF5, thereby contributing to hepatic IRI (HIRI). In cardiac IRI, activation of the JAK2/STAT3 signaling pathway upregulates IRF5, which, in turn, promotes the transcription of VCAM-1. In cerebral IRI models, DJ-1 disrupts the interaction between p62 and TRAF6, thereby blocking the nuclear translocation of IRF5. The IRF9/Sirt1/p53 axis-mediated activation of cellular injury represents a key regulatory pathway in IRI.

Despite this, IRF-9 has been found to potentially act as a “defender” in several important organ IRI models (**Figure 6**). The researchers conducted a warm liver IRI model using IRF-9 KO mice and demonstrated that IRF-9 KO significantly reduced the area of liver parenchymal necrosis and immune cell infiltration after warm liver IRI. It was also observed that IRF-9 KO alleviated liver IRI by restoring liver function and reducing the release of inflammatory mediators. Through dual-luciferase reporter gene and deacetylase activity analysis, it was shown that IRF-9 directly suppressed the promoter activity of Sirt1; however, it increased the acetylation level of p53. The two biological effects involved IRF9, synergistically enhancing the level of hepatocyte apoptosis during the liver IRI process (150). In the MIR model, IRF-9 was also significantly upregulated and mediated more serious myocardial cell death and inflammatory levels. For the MIR treatment after IRF-9 KO, the left ventricular contractile function and damage level of the mice’s hearts were significantly improved. Mechanistically, IRF-9 exacerbated myocardial cell injury with a negative regulatory pattern of the Sirt1–p53 axis (151). Similarly, through whole-genome mRNA microarray analysis after cerebral IRI between wild-type and IRF-9 KO mice, it was found that IRF-9 directly mediated neuronal death, and the discovery revealed that deacetylase Sirt1 was a downstream target of IRF-9. Mechanistically, IRF-9 downregulated the activity of deacetylase Sirt1, leading to the activation of p53-mediated cell death signals (152). A recent study has indicated that the long non-coding RNA TTTY15 could act as a sponge to suppress the expression of mir-520a-3p, thereby promoting the neuro-apoptosis induced by brain IRI through the mediation of IRF-9 (153). These findings suggested that the upstream non-coding RNA of IRF-9 is a prospective target to regulate IRF-9 expression. In conjunction with the aforementioned results, it is not difficult to discern that IRF-9 is a responsive stimulus factor for tissue IRI, and the cell damage activation mediated by the IRF-9/Sirt1/p53 axis is crucial. The targeted inhibition of both IRF-9 and its induced upstream or downstream network will provide more ideas for the prevention and treatment of tissue IRI.

## Progress in drug development targeting IRFs

IRFs, serving as core regulatory molecules of inflammation, oxidative stress, and cell death, have increasingly attracted attention for their role in IRI (6). Recently, significant progress has been made in drug development targeting IRFs and their related signaling pathways (**Table 1**)—for example, these drugs’ protective effects against multi-organ IRI by intervening in the nuclear translocation of IRFs have been demonstrated via regulating upstream/downstream signaling networks and modulating cell death patterns (172).

**Table 1:** Advances in the development of therapeutic agents targeting the IRFs and associated signaling pathways.

Compound	Model	Involved signaling pathways	Main effects and outcomes
Anethole (154)	Hepatic IRI in mice	Reduced both nuclear translocation of IRF-1 and IRF-1/HMGB1/TLR signaling	Decreased pro-inflammatory factors and alleviated HIRI
Chlorogenic acid (155)	Hepatic IRI in mice	Reduced IRF-1/HMGB1/TLR4/NF-κB signaling	Decreased oxidative stress, mitochondrial damage, and apoptosis
Pectolinarigenin (156)	Hepatic IRI in mice	Activated the PI3K/AKT/Nrf2 signaling pathway	Inhibited oxidative stress, inflammatory response, and apoptosis
Melatonin (157)	Hepatic IRI in mice	Downregulated the NF-κB signaling pathway	Alleviated the inflammatory response, protected the liver from ischemia–reperfusion injury
Cordycepin (158)	Hepatic IRI in mice	Inhibited the MAPK/NF-κB signaling pathway	Attenuated the inflammatory response and the production of pro-apoptosis proteins
Dexmedetomidine (159)	Cerebral IRI in rats	Downregulated IRF5/IRF7 expression and regulated the TLR4/MyD88/NF-κB signaling pathway	Reduced blood–brain barrier disruption and neuroinflammation
TAK-242 (160)	Renal IRI in rats	Inhibited the TLR4/TRIF/IRF3 signaling pathway	Reduced IFN-β production, renal tubular necrosis, and pro-inflammatory cytokine secretion
Melatonin (161, 162)	Hepatic IRI in rats	Regulated the JAK2/STAT1 signaling pathway	Inhibited IRF1 nuclear translocation and reduced markers of oxidative stress (MDA) and liver enzymes (ALT/AST)
Curcumin (163, 164)	Myocardial IRI in mice	Regulated IRF4/IRF8 and activated the PI3K/Akt/mTOR signaling pathway	Upregulated IRF4-mediated anti-apoptotic pathway, attenuated oxidative stress, inhibited apoptosis, and reduced myocardial infarct size
Necrostatin-1 (165, 166)	Intestinal IRI in mice	Inhibited the RIPK1/RIPK3/MLKL signaling pathway	Blocked necroptotic apoptotic pathway and inhibited IRF2-driven intestinal epithelial barrier damage
Baicalein (167, 168)	Cerebral IRI in rats	Activated the AMPK signaling pathway and inhibited NLRP3/Caspase-1/Gasdermin-D pathway	Decreased inflammatory response and cellular pyrokinesis, inhibited NLRP3 inflammatory vesicle activation, and improved neurological function score
Rapamycin (169, 170)	Cardiac IRI in mice	Inhibited the mTOR signaling pathway	Regulated IRF1/IRF8 expression, inhibited macrophage M1 polarization through the mTOR–IRF1 axis, attenuated myocardial fibrosis, and ameliorated myocardial ischemia–reperfusion injury
Resveratrol (171)	Cardiac IRI in mice	Activated the SIRT1 signaling pathway and inhibited NF-κB activity	Inhibited IRF1/IRF3 activation and reduced cardiomyocyte apoptosis and inflammatory factor (TNF-α, IL-6) release

In the field of hepatic IRI, natural products that directly target IRF-1 have been most extensively investigated. Anethole, as the first compound confirmed to possess IRF-1 inhibitory activity, exerts hepatoprotective effects through dual mechanisms: on one hand, it blocks the nuclear translocation of IRF-1 and suppresses its transcriptional activity; on the other hand, it interferes with the HMGB1/TLR signaling axis, reducing the cascading release of downstream pro-inflammatory factors (154). This discovery provided crucial preliminary clues for subsequent research, namely, that inhibiting the nuclear localization of IRF-1 could effectively ameliorate HIRI. Building upon this foundation, chlorogenic acid further expanded the regulatory depth of this pathway—it not only suppresses the IRF-1/HMGB1/TLR4/NF-κB signaling axis but also possesses direct antioxidant and mitochondrial protective functions, forming a triple protective network of “anti-inflammatory, antioxidant, and anti-apoptotic” effects (155). Notably, pectolinarigenin represents a strategy of indirect IRF-1 modulation, which improves cellular redox status through activating the PI3K/AKT/Nrf2 antioxidant pathway, thereby feedback-inhibiting excessive IRF-1 activation (156), suggesting the existence of cross-talk between metabolic regulation and IRF suppression. Melatonin, as the most extensively studied endogenous molecule, exhibits multi-pathway regulatory characteristics in HIRI: it can reduce the IRF-1-coordinated transcription of inflammatory genes through inhibiting NF-κB signaling, block type I interferon signal amplification through the JAK2/STAT1 pathway, and maintain mitochondrial homeostasis through the PGAM5–mPTP axis (157), demonstrating the close association between IRF regulation and organelle protection. Cordycepin indirectly modulates IRF-1-mediated inflammatory responses through the dual inhibition of MAPK/NF-κB while simultaneously reducing pro-apoptotic protein expression (158); however, as an adenosine analogue, its direct effect upon IRF-1 remains to be elucidated.

Drug development for cerebral IRI exhibits the characteristics of targeting distinct IRF members. Dexmedetomidine, a clinically employed sedative, has been found to downregulate the expression of IRF5 and IRF7, reduce excessive type I interferon production, and protect the blood–brain barrier integrity through the TLR4/MyD88/NF-κB pathway, providing novel evidence for perioperative neuroprotection (159). Baicalin, through activating AMPK signaling to inhibit NLRP3 inflammasome assembly, simultaneously modulates IRF1/IRF8 expression to suppress macrophage M1 polarization, revealing the integrated role of metabolic-immune-IRF regulatory networks in neuroprotection (167, 168).

Research into myocardial IRI has highlighted the close association between IRF regulation and metabolic reprogramming. Curcumin influences macrophage polarization status through modulating IRF4/IRF8 while activating the PI3K/Akt/mTOR and Sirt1/AKT/FoxO3a pathways to reduce cardiomyocyte apoptosis and ferroptosis (163, 164). Rapamycin, as an mTOR-specific inhibitor, feedback-regulates mitochondrial respiration and intrinsic apoptosis through the mTOR–IRF1 axis and suppresses macrophage M1 polarization (169, 170); its existing applications in organ transplantation facilitate clinical translation. Resveratrol demonstrates particular value under metabolic aberrant conditions, alleviating obesity-related oxidative stress and inflammatory injury through inhibiting IRF1/IRF3 activation and promoting SIRT1/SIRT3 activity, suggesting that IRF-targeting strategies directed at specific metabolic phenotypes may possess greater clinical advantages (171). Investigations into renal and intestinal IRI have expanded the application scenarios of IRF-targeted drugs. TAK-242, as a TLR4-specific inhibitor, reduces IFN-β production and tubular necrosis by blocking the TLR4/TRIF/IRF3 signaling pathway (160), representing an efficient strategy of indirect IRF3 inhibition from upstream nodes. Although Necrostatin-1 primarily blocks necroptosis through inhibiting RIPK1/RIPK3/MLKL signaling, its effect in reducing IRF2-dependent intestinal epithelial cell death (165, 166) hints at complex interactions between programmed cell death modalities and IRF regulation.

However, the main limitation of drug development lies in the fact that the majority of studies have been conducted in animal models and are still at the basic research stage, lacking large-scale clinical trials to verify their efficacy and safety. Therefore, caution should be exercised when extrapolating the research results from animal models to humans. Although some signaling pathways and molecular mechanisms related to IRFs have been identified, their detailed roles in IRI are still not fully understood—for instance, the interactions between IRFs and other signaling molecules, as well as the functional differences of IRFs in different cell types, and the role of IRFs in multi-organ cross-tissue IRI require further investigation.

In the future, investigational therapies targeting IRFs may be utilized in combination with other treatment modalities, potentially yielding synergistic effects—for instance, anti-inflammatory agents could be combined with antioxidants to simultaneously suppress inflammatory responses and oxidative stress. Such combinatorial therapeutic strategies may allow a more comprehensive targeting of the multiple pathophysiological mechanisms underlying IRI, thereby enhancing treatment efficacy. Furthermore, therapeutic development targeting IRFs in IRI is evolving from the exploration of single molecular entities toward an integrated, multi-technology platform paradigm. From the perspective of precise regulation of IRF expression: complementary nucleic acid sequences can be designed to degrade or inhibit the mRNA of specific IRF genes, achieving the translational knockdown of pro-inflammatory IRF proteins (173). Additionally, the CRISPR–Cas9 system enables permanent genomic DNA editing, allowing the complete knockout of deleterious IRF genes or correction of pathogenic single nucleotide polymorphisms (SNPs) via base editing or prime editing technologies (174). To enhance drug targeting precision: synthetic or biologically derived nanoparticles (e.g., liposomes, polymeric nanoparticles, exosomes) can be engineered as drug carriers (175). By modulating their size, surface charge, and functionalization (e.g., conjugating targeting peptides or antibodies specific to certain organs or cell types), spatially specific drug accumulation at pathological sites can be achieved. Alternatively, artificial intelligence (AI) can be employed to analyze multi-omics data (genomic, transcriptomic, proteomic) to predict critical IRF isoforms and their previously unknown upstream and downstream networks in IRI, thereby identifying novel drug targets (176). AI-facilitated rational design may further enable the development of novel small-molecule or peptide inhibitors that specifically bind IRF proteins and disrupt their functions (e.g., dimerization, DNA binding). This integrated approach holds promise for overcoming current research limitations and may ultimately offer new hope for patients suffering from IRI.

## Conclusion

Tissue IRIs, a clinical syndrome triggered by a variety of etiological factors, involves a rapid derangement of various pathophysiological factors during the process of ischemia, followed by reperfusion of blood reflow and recirculation. The activation of multiple pathogenic factors and associated pathways within this process is a cluster of crucial elements to evoke tissue IRIs. As a vital regulatory factor superfamily, IRFs have been generally researched to contribute to an essential role in human diseases, including the field of tissue IRIs. Given the structural and functional differences among various IRFs, as well as the distinct pathways they are involved in, there is a certain heterogeneity in mediating specific tissue IRIs. To date, the majority of IRF family members have been reported to take part in tissue IRIs, but the regulatory function of IRF-7 in tissue IRI remains to be unclarified. Interestingly, our team’s latest work will seemingly fill the gap of IRF7 regulation mechanism in LTx and IRI. Despite this, recent research has still found that IRF-7 might be referred to the recovery of neurological function following hypothermic traumatic brain injury (177). In another study, combining bioinformatics analysis with *in vivo* verification, IRF-7 was surprisingly found to be downregulated in intestinal ischemia–reperfusion injury (178). These findings suggested that including IRF7, IRFs ought to be regarded as the “star element” in tissue IRI research and still required further investigation to reveal.

**Figure 7:**
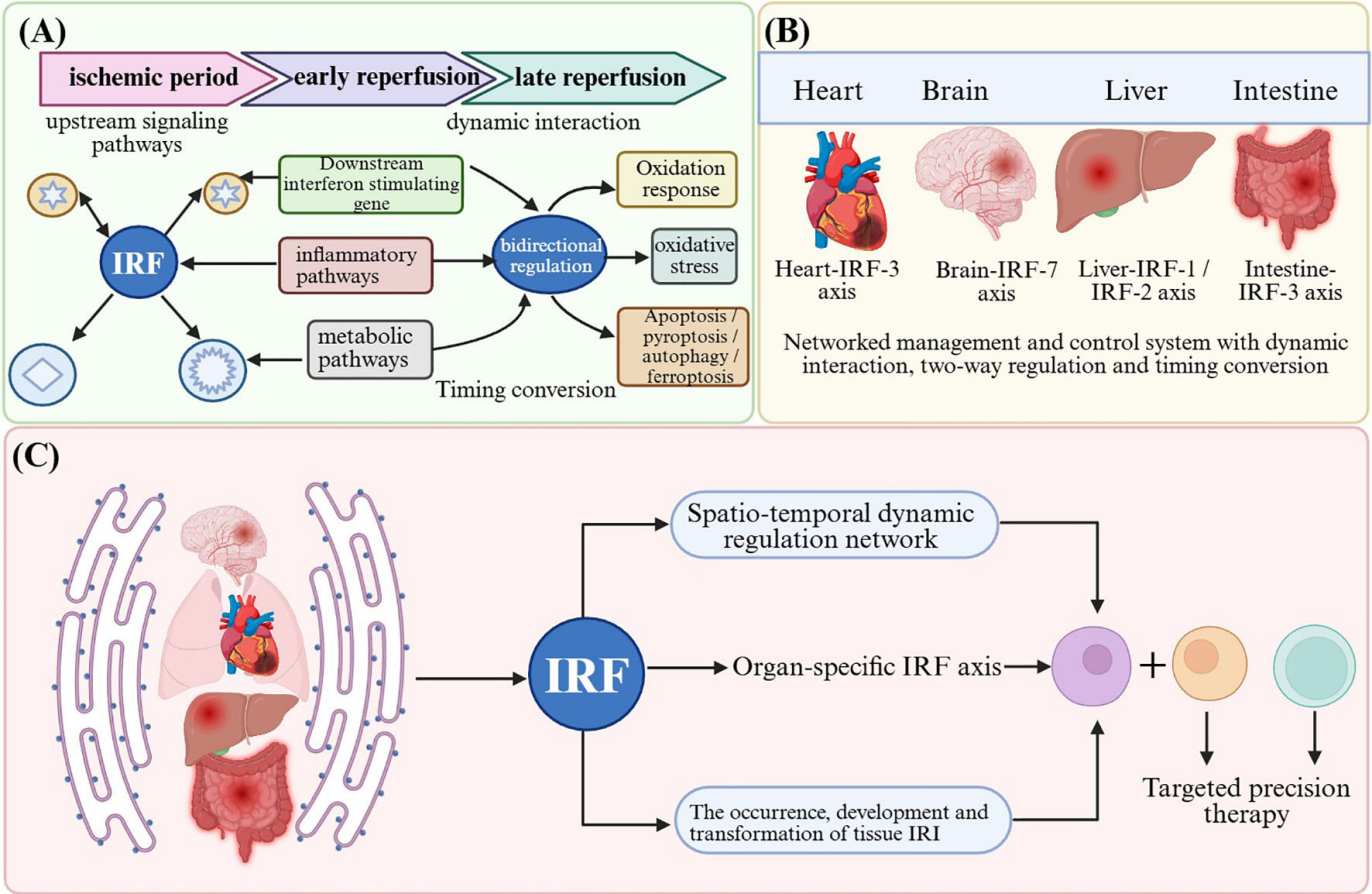
Schematic diagram of the “dynamic regulatory network” and “organ–IRF axis” mediated by IRFs in tissue IRI. **(A)** IRF dynamic regulatory network: With IRF family members as core nodes, dynamic interactions, bidirectional regulation, and temporal switching are formed with upstream signaling pathways, downstream interferon-stimulated genes, inflammatory pathways, metabolic pathways, oxidative stress, and multiple cell fate program including apoptosis, pyroptosis, autophagy, and ferroptosis across distinct chronological stages of IRI, including the ischemic phase, early reperfusion and late reperfusion. **(B)** Organ-IRF axis: Different organs (heart, brain, liver, intestine, etc.) establish organ-specific regulatory axes centered on particular IRFs, determined by their unique cellular composition, immune microenvironment and pathological characteristics. **(C)** Integrated model: IRFs collectively determine the initiation, progression and resolution of tissue IRI through the spatiotemporal dynamic regulatory network and organ-specific IRF axes, providing theoretical foundations for targeted precision therapeutics.

This review integrates the latest research progress of IRFs in tissue IRI, not only revealing the molecular basis of their functional heterogeneity but also putting forward two innovative theoretical frameworks: “dynamic regulatory network” and “organ–IRF axis” (**Figure 7**). The core of the “dynamic regulatory network” theory lies in dismantling the traditional linear perception of signaling pathways instead of regarding IRFs as highly plastic regulatory hubs across spatiotemporal dimensions (1, 2). Specifically, during the progression of hepatic IRI, the function of IRF1 is not static: in the early ischemic phase (0–30 min), IRF-1 undergoes rapid phosphorylation and nuclear translocation to initiate pro-inflammatory gene transcription, and at the peak reperfusion stage (2–6 h), IRF-1 amplifies inflammatory signals through positive feedback loops (179), while during the repair phase (after 24 h), the post-translational modification pattern of IRF-1 shifts, with ubiquitin-mediated degradation signals becoming dominant and inflammatory responses gradually subsiding (180). This dynamic process explains why simple IRF-1 inhibition may not represent the optimal strategy and why temporally specific precision regulation proves more critical. Furthermore, the functional output of IRF-3 depends strictly upon its subcellular localization: when localized to the mitochondria, IRF-3 cooperates with BAX to release cytochrome c and trigger apoptosis (181). Following translocation to the nucleus, IRF-3 activates type I interferon genes to initiate antiviral-like immune responses (3), whereas in the cytoplasm, IRF-3 may form complexes with STING to sense cytosolic DNA signals (5). This precise “localization–function” correspondence enables the same molecule to exert diametrically opposite biological effects under distinct spatiotemporal contexts. More importantly, IRFs do not function in isolation but rather form complex feedback networks with NF-κB, MAPK, JAK-STAT, and other pathways (182)—for instance, in cardiac IRI, activation of IRF-3 can simultaneously suppress NF-κB signaling (through competitive binding to IKKβ), and such interactions render simple activation or inhibition of any single pathway liable to produce unpredictable network effects (183). The dynamic regulatory network theory emphasizes that IRFs must be understood within the topology of the overall network rather than studied in isolation.

The “organ–IRF axis” theory reveals highly selective regulatory relationships between specific IRF members and target organs, shaped collectively by cellular composition, metabolic characteristics, and immune microenvironment of each organ (57, 129). The hepatic–IRF axis represents “dual metabolic-immune regulation”. As a metabolic hub, the liver’s abundant mitochondrial content enables the mitochondrial translocation of IRF-1 (184); simultaneously, the liver’s unique sinusoidal structure allows Kupffer cells to rapidly sense circulating DAMPs and activate IRF-3. The defining feature of this axis is the “competitive balance” between IRF-1 and IRF-2, with IRF-1 dominating pro-inflammatory injury and IRF-2 exerting protective effects through competitive DNA binding (185). In drug development, anethole and chlorogenic acid achieve hepatoprotective effects precisely by intervening in the IRF-1 nuclear translocation step within this axis (154, 155). The cardiac–IRF axis constitutes an “energy crisis response”. The high energy demands of cardiomyocytes render them particularly sensitive to mitochondrial localization of IRF-3. Unlike the liver, IRF-3 activation in cardiac IRI directly triggers mitochondrial apoptotic programs rather than primarily relying upon IRF-1 (186). Rapamycin modulates mitochondrial function through inhibiting mTOR-IRF-1 axis feedback (169, 170), while resveratrol targets dual IRF1/IRF3 activation in the context of obesity (171), reflecting the metabolic sensitivity of this axis. The cerebral–IRF axis represents a “neuroimmune barrier”. The integrity of the blood–brain barrier determines the spatiotemporal window of IRF activation (187). Dexmedetomidine downregulates IRF5/IRF7 expression and baicalin modulates IRF1/IRF8, both targeting the specialized immune populations of microglia and astrocytes rather than acting directly upon neurons (159, 168). The uniqueness of this axis lies in the association between IRF regulation and neurological functional recovery, namely, that IRF-7 activation levels correlate positively with neurological recovery following traumatic brain injury under hypothermia. The renal–IRF axis demonstrates “filtration–reabsorption sensitivity”. The high metabolic activity of renal tubular epithelial cells renders them sensitive targets for IRF-1-mediated apoptosis (188). TAK-242 indirectly suppresses IRF3 activation by blocking the upstream TLR4/TRIF/IRF3 signaling node, reducing tubular necrosis and IFN-β production (160) and exemplifying a strategy of upstream regulation of this axis. The intestinal–IRF axis displays “microbiota-dependent regulation”. The presence or absence of intestinal microbiota determines the functional polarity of IRF-3. Under germ-free conditions, IRF-3 promotes NET formation and exacerbates injury; in the presence of microbiota, microbial metabolites suppress excessive IRF-3 activation (102, 189). Necrostatin-1 indirectly reduces IRF2-dependent intestinal epithelial cell death by inhibiting necroptosis (165), suggesting tight coupling between programmed cell death modalities and IRF regulation within this axis.

In summary, single-cell sequencing can reveal the cell type specificity of organ–IRF axes (129), organoid models can simulate the temporal dynamics of regulatory networks (55), and artificial intelligence prediction can integrate multidimensional data to optimize drug design (58). Future drug development must integrate these technologies to achieve the transition from “discovering effective compounds” to “designing intelligent drugs”—for instance, developing organ-specific delivery systems (such as liver-targeted IRF-1 nuclear translocation inhibitors or cardiac mitochondria-localized IRF-3 modulators) (190) or exploring the combined application of gene editing (such as CRISPR-mediated IRF2 overexpression) with existing drugs (191) will all propel critical steps forward in translating research findings from this field into clinical practice.
